# Deep learning algorithm for the automated detection and classification of nasal cavity mass in nasal endoscopic images

**DOI:** 10.1371/journal.pone.0297536

**Published:** 2024-03-13

**Authors:** Kyung Won Kwon, Seong Hyeon Park, Dong Hoon Lee, Dong-Young Kim, Il-Ho Park, Hyun-Jin Cho, Jong Seung Kim, Joo Yeon Kim, Sang Duk Hong, Shin Ae Kim, Shin Hyuk Yoo, Soo Kyoung Park, Sung Jae Heo, Sung Hee Kim, Tae-Bin Won, Woo Ri Choi, Yong Min Kim, Yong Wan Kim, Jong-Yeup Kim, Jae Hwan Kwon, Myeong Sang Yu

**Affiliations:** 1 Department of Otolaryngology, Samsung Changwon Hospital, Sungkyunkwan University School of Medicine, Changwon, Korea; 2 Department of Biomedical Informatics, College of Medicine, Konyang University, Daejeon, Korea; 3 Department of Otolaryngology-Head and Neck Surgery, Chonnam National University Medical School & Hwasun Hospital, Hwasun, Korea; 4 Department of Otorhinolaryngology-Head and Neck Surgery, Seoul National University Hospital, Seoul, Korea; 5 Department of Otorhinolaryngology-Head and Neck Surgery, Korea University Guro Hospital, Korea University College of Medicine, Seoul, Korea; 6 Department of Otorhinolaryngology, Gyeongsang National University School of Medicine and Gyeongsang National University Hospital, Jinju, Korea; 7 Department of Otorhinolaryngology-Head and Neck Surgery, College of Medicine, Jeonbuk National University, Jeonju, Korea; 8 Department of Otolaryngology-Head and Neck Surgery, Kosin University College of Medicine, Busan, Korea; 9 Department of Otorhinolaryngology–Head and Neck Surgery, Samsung Medical Center, Sungkyunkwan University School of Medicine, Seoul, Korea; 10 Department of Otolaryngology-Head and Neck Surgery, Soonchunhyang University Seoul Hospital, Soonchunhyang University College of Medicine, Seoul, Korea; 11 Department of Otorhinolaryngology-Head and Neck Surgery, Dankook University College of Medicine, Cheonan, Korea; 12 Department of Otorhinolaryngology-Head and Neck Surgery, Chungnam National University Sejong Hospital, College of Medicine, Sejong, Korea; 13 Department of Otorhinolaryngology-Head and Neck Surgery, School of Medicine, Kyungpook National University Chilgok Hospital, Kyungpook National University, Daegu, Korea; 14 Department of Otorhinolaryngology-Head and Neck Surgery, National Medical Center, Seoul, Korea; 15 Department of Otorhinolaryngology‒Head and Neck Surgery, Seoul National University Bundang Hospital, Seongnam, Korea; 16 Department of Otorhinolaryngology–Head and Neck Surgery, College of Medicine, Chungnam National University, Daejeon, Korea; 17 Department of Otorhinolaryngology, Inje University Haeundae Paik Hospital, Busan, Korea; 18 Department of Otorhinolaryngology-Head and Neck Surgery, Asan Medical Center, University of Ulsan College of Medicine, Seoul, Korea; University of Catania, ITALY

## Abstract

Nasal endoscopy is routinely performed to distinguish the pathological types of masses. There is a lack of studies on deep learning algorithms for discriminating a wide range of endoscopic nasal cavity mass lesions. Therefore, we aimed to develop an endoscopic-examination-based deep learning model to detect and classify nasal cavity mass lesions, including nasal polyps (NPs), benign tumors, and malignant tumors. The clinical feasibility of the model was evaluated by comparing the results to those of manual assessment. Biopsy-confirmed nasal endoscopic images were obtained from 17 hospitals in South Korea. Here, 400 images were used for the test set. The training and validation datasets consisted of 149,043 normal nasal cavity, 311,043 NP, 9,271 benign tumor, and 5,323 malignant tumor lesion images. The proposed Xception architecture achieved an overall accuracy of 0.792 with the following class accuracies on the test set: normal = 0.978 ± 0.016, NP = 0.790 ± 0.016, benign = 0.708 ± 0.100, and malignant = 0.698 ± 0.116. With an average area under the receiver operating characteristic curve (AUC) of 0.947, the AUC values and F1 score were highest in the order of normal, NP, malignant tumor, and benign tumor classes. The classification performances of the proposed model were comparable with those of manual assessment in the normal and NP classes. The proposed model outperformed manual assessment in the benign and malignant tumor classes (sensitivities of 0.708 ± 0.100 vs. 0.549 ± 0.172, 0.698 ± 0.116 vs. 0.518 ± 0.153, respectively). In urgent (malignant) versus nonurgent binary predictions, the deep learning model achieved superior diagnostic accuracy. The developed model based on endoscopic images achieved satisfactory performance in classifying four classes of nasal cavity mass lesions, namely normal, NP, benign tumor, and malignant tumor. The developed model can therefore be used to screen nasal cavity lesions accurately and rapidly.

## Introduction

Tumorous lesions in the nasal cavity and paranasal sinuses are of several histopathological types and are highly heterogeneous [1–3]. Non-neoplastic and neoplastic lesions are common in the nasal cavity [2]. The majority of nasal cavity mass lesions are non-neoplastic, such as nasal polyps (NPs), which are typically bilateral and multiple and have a prevalence of 1%–4% [4, 5]. Inverted papillomas (IPs) are the most common benign tumor occurring in the nasal cavity, consisting of 0.5%–0.7% of all nasal cavity tumors and seldom exhibit malignant features [6]. Malignant tumors of the nasal cavity are rare and account for only 3% of all head and neck tumors and less than 1% of all malignancies [7]. Treatment strategies differ according to the histopathologic type of the tumorous lesion. Generally, medical therapy is the preferred treatment for NPs, and complete resection through an endoscopic approach is used for IPs. However, malignant tumors of the nasal cavity require multiple treatment modalities including surgery, radiation, and chemotherapy. Therefore, differentiating nasal cavity tumors is critical before determining treatment strategies.

In clinical practice, nasal endoscopy is routinely performed to visualize and discriminate various conditions in the nasal cavity, which is the first step in the diagnosis of nasal cavity tumors [8, 9]. A nasal endoscopy followed by an endoscopically directed biopsy at the suspicious area is crucial for the confirmation of nasal cavity mass lesions including benign or malignant tumors. If the appearance of the mass lesions is neoplastic, clinicians perform a biopsy under nasal endoscopy. However, clinically differentiating non-neoplastic and neoplastic lesions in the nasal cavity is difficult because of the heterogeneity of gross appearance and continuum of disease severity [6]. Manual visual observation of nasal cavity lesions from endoscopic images is subjective and depends on the experience of the examiner. Although histopathological diagnosis is the gold standard for diagnosing nasal cavity tumors, the process is time-consuming, and limitations exist in performing biopsy in all patients with nasal cavity mass lesions in clinical settings in terms of time, cost, or patient’s physical condition. Therefore, faster and consistent screening methods are required for reducing individual variability for making early decisions and allowing next-step evaluations such as biopsy or imaging analyses for suspicious cases.

Computer-assisted image recognition algorithms can be used to detect and classify lesions from medical images for improving diagnosis. Convolutional neural networks (CNNs), a popular deep learning algorithm, have exhibited excellent image classification performance [10, 11]. Studies have revealed that a CNN can exhibit comparable results or outperform manual clinical visual assessment (CVA) in the diagnosis of diseases such as retinopathy [12], gastrointestinal disease [13, 14], skin malignancy [15], and laryngeal disease [16]. A previous preliminary study demonstrated that CNN-based algorithms can serve as a reliable model for distinguishing normal nasal cavities, IP lesions, and NP lesions using nasal endoscopic images, achieving accuracies of 0.81±0.14, 0.57±0.07, and 0.83±0.21, respectively [17]. While this study included three categories, only two types of tumorous lesions (IP and NP) were considered, and it was limited by the size of the dataset (100 images for each class). To the best of our knowledge, no studies have developed models to identify a range of diagnostic entities including various benign or malignant nasal cavity tumors beyond IP and NP lesions. An automatic diagnostic system using a CNN model could be a practical tool for the screening of nasal cavity tumors, although the model could not completely replace manual clinical assessments. In this study, a CNN-based computer-aided diagnosis system was proposed for the automatic detection and classification of nasal cavity tumors, including NP, benign tumors, and malignant tumors. The performance of the model and its clinical applicability were validated. This study marks a significant step forward in applying deep learning to the classification of nasal cavity mass lesions, potentially enhancing diagnostic speed and accuracy. The key contributions include the following:

• This study represents a novel attempt to utilize deep learning for the classification of a wide range of nasal endoscopic findings, including normal nasal cavities, NPs, benign tumors, and malignant tumors.
• A large-scale, multi-institutional, biopsy-confirmed dataset from 17 medical centers was constructed to develop the diagnostic model.
• We compared the performance of deep learning-based classification to the expertise of medical professionals in effectively differentiating between each class, as well as between urgent (malignant) and nonurgent lesions.
• Our paper discusses the challenges tied to the unique characteristics of nasal endoscopy images, the limitations of the current approach, and promising directions for future research.

## Materials and methods

### Preparation of nasal endoscopic images

Nasal endoscopic images and clinicopathological data of patients who had undergone routine clinical screening for sinonasal diseases were obtained from 17 healthcare centers in South Korea. Nasal endoscopic images were obtained from 2074 patients with a normal nasal cavity, 1533 cases of NPs (NP class), 958 cases of benign tumors (benign class), and 533 cases of malignant tumors (malignant class), all pathologically confirmed, yielding a total of 5098 images. The images were in PNG or JPG format with three color channels, namely red, green, and blue, and were captured using a rigid 4-mm nasal endoscope and an endoscopic capture recorder with widths and heights ranging from 300 to 600 pixels. After excluding 328 low-quality images with issues such as blurring; inadequate lighting; instrument interference; or lesion obscuration by foreign substances, crust, or purulent discharge, a dataset (*N* = 4770) was constructed using endoscopic images of 1963 normal cases, 1,442 NPs, 847 benign tumors, and 818 malignant tumor lesions. Next, 4340 images were randomly partitioned into training and validation sets at a 3:2 ratio to develop the algorithm. The remaining 400 images were used for the test set such that 100 images were obtained per pathologic class. These images were used to compare the performance of the CNN-based algorithm to the CVAs by otolaryngologists. This study was approved by the Ethics Committee of Asan Medical Center, and the need for informed consent was waived owing to the retrospective design of the study.

### Data preprocessing and augmentation

Before developing the deep learning model, the images were preprocessed to standardize the resolution, extension, and size of collected images. All code and endoscopic image samples are accessible on GitHub (https://github.com/shpark5779/SNT_Research/tree/main). First, for consistency among nasal endoscopic images, each JPG image file was converted into PNG format. Some raw nasal endoscopic images contained black circular edges with artifacts or text on the background, such as date and patient information. Such artifacts can cause considerable data bias when training a deep learning model. Therefore, background noise and useless parts were removed from the images as follows:1) The black circle contour was identified using an Open CV function called minEnclosingCircle, and its outside part was removed. 2) The NumPy function “zeros” was used to create a black array as the background, and the target part (inside the black circle contour) was placed on it. 3) These images were cropped to a standard region based on the black circle contour using the Open CV function called boundingRect. The images were resized to 224 × 224 or 331 × 331 pixels using the nearest-neighbor interpolation of the required input dimensions for the models.

**Fig 1:**
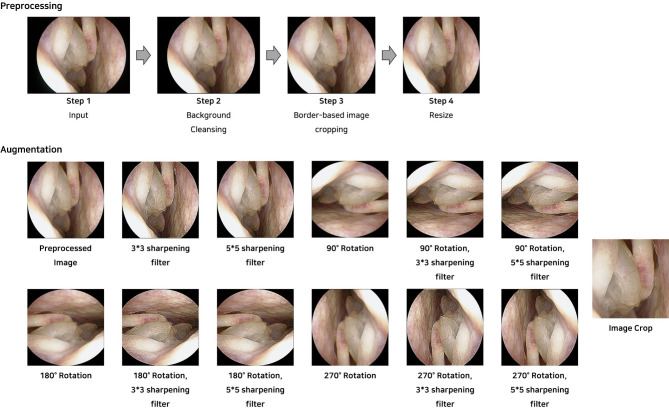
Image preprocessing and augmentation steps on the nasal endoscopic images.

In this study, the number of images was insufficient for training the high-dimensional parameters of the CNN model. For data augmentation, two types of sharpening filters (3 × 3 or 5 × 5 pixels) were applied to benign and malignant tumor images, which were rotated at 90°, 180°, and 270° and cropped manually. A 3 × 3 sharpening filter was applied to process the NP images with rotation and cropping, whereas the normal class images were not cropped (Fig 1). The total training and validation dataset consisted of 149,043 normal nasal cavity, 311,043 nasal polyp, 9,271 benign tumor, and 5,323 malignant tumor lesion images.

### CNN training for classification

In this study, transfer learning was performed using a CNN model on the ImageNet database (1.2 million images with 1000 categories) was performed. The categorical cross-entropy function was used as the loss function for all models. Thus, the labels were created in one-hot encoding and entered into the model. The categorical cross-entropy value (*L_CE_*) is computed as follows:

$$L_{CE}=-\sum_{i=1}T_{i}\log\left(S_{i}\right),for\;n\;classes$$

where *T_i_* is the label and S_i_ is the value of the softmax function.

The weights for all layers except for the top layer were initialized using weights from the pretrained model. To avoid class imbalance and overfitting problems, particularly in the benign and malignant tumor classes, various class weights were applied according to the class, as displayed in the following formula:

$$W_{i}=\frac{1}{{Number\;of\;Class}_{i\;}image}\frac{Number\;of\;Total\;image}{Number\;of\;Class}$$

**Fig 2:**
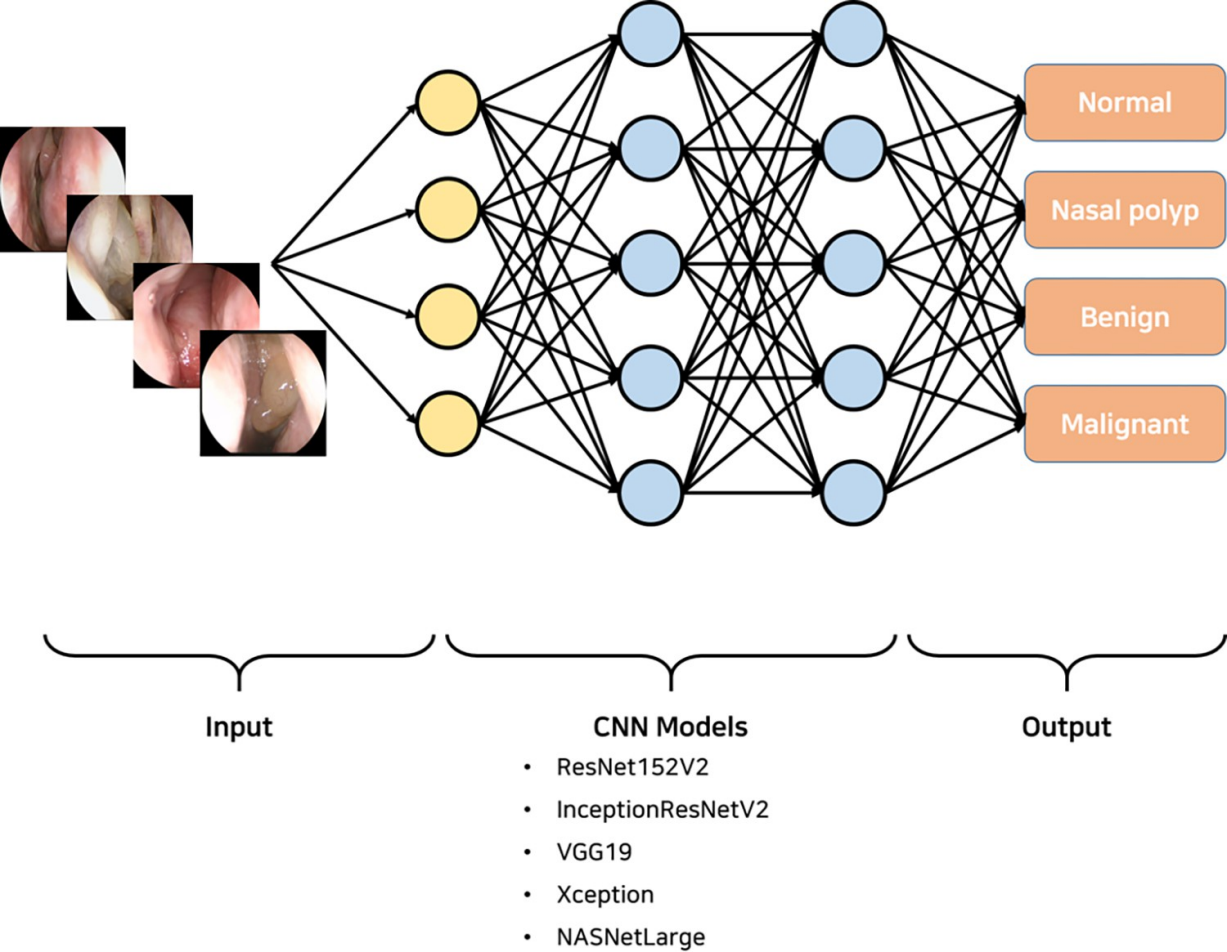
Proposed deep learning architecture and classification pipeline. The detection algorithm is a model based on the deep CNN. Preprocessed images are input to the pretrained CNN model to determine the class that the image belongs to, namely normal, NP, benign, and malignant tumors.

**Fig 3:**
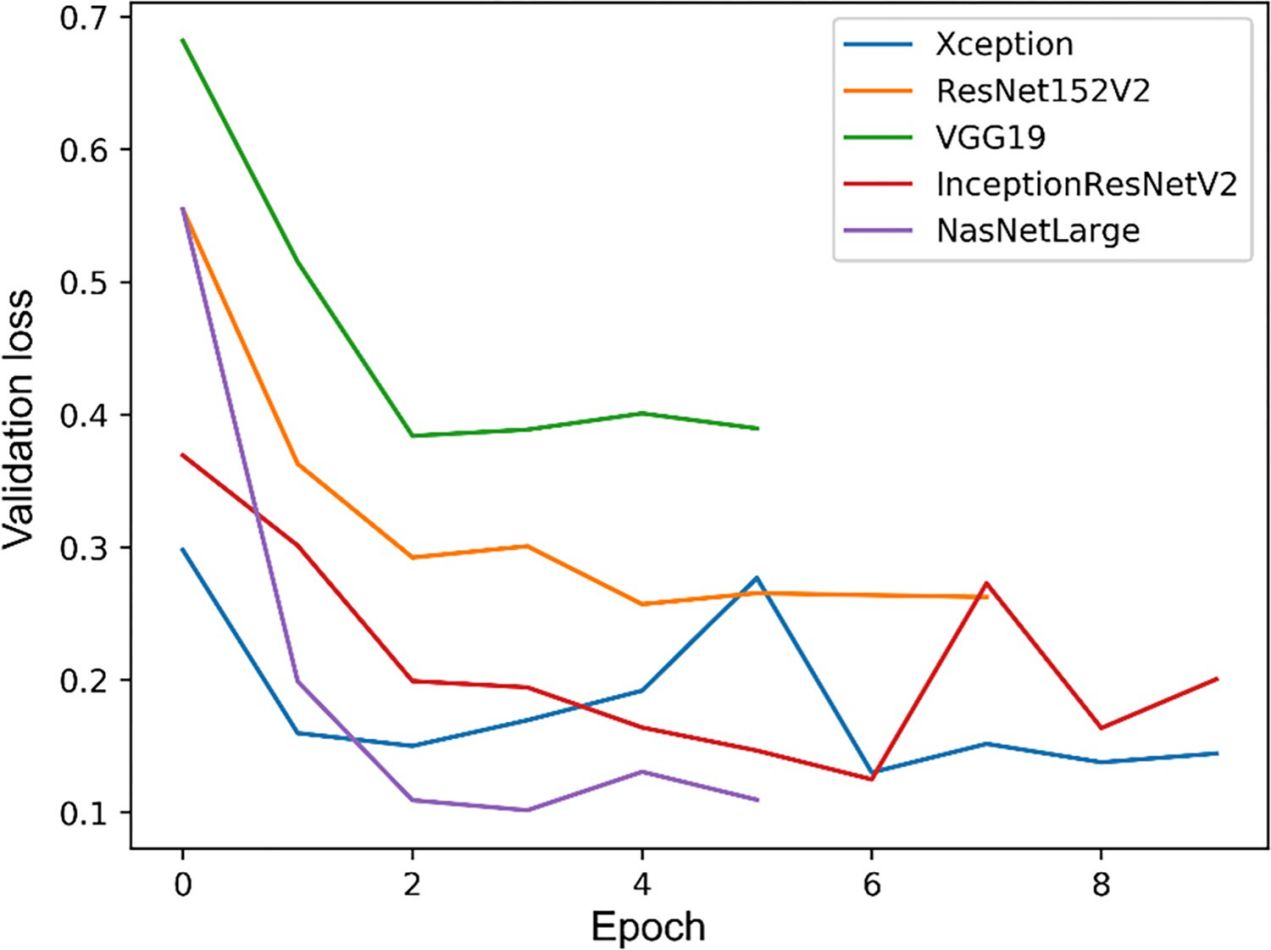
Changes in the loss function value on the validation set.

A CNN architecture was constructed to calculate the probability of each pathological class of endoscopic images by using VGG19, +ResNet152V2, InceptionResNetV2, NasNetLarge, and Xception (Fig 2). To use these neural network models, Keras, an open-source machine learning framework, was used as an interface for the TensorFlow library. The validation process was conducted using the randomly partitioned validation set to improve model performance. An early termination method was applied to reduce the risk of overfitting; the training was terminated when the value of the loss function did not change by more than 0.01 during three epochs (Fig 3). Using the GridSearchCV method, all possible combinations of each hyperparameter were searched to determine the best model and combination of values yielding the best score. The final combination of hyperpatameter values were as follows:

- VGG19: batch size = 10, learning late = 1.00E-05
- ResNet152V2: batch size = 10, learning late = 1.00E-05
- InceptionResNetV2: batch size = 30, learning late = 0.0001
- NasNetLarge: batch size = 10, learning late = 1.00E-05
- Xception: batch size = 20, learning late = 0.0001

Image preprocessing and CNN training were performed using Python(3.8.11) and the TensorBoard (2.9.0), TensorFlow (2.4.1), Keras (2.4.3), scikit-learn (1.0.2), NumPy (1.20.3), and OpenCV (4.0.1) Python packages.

### Performance evaluation

**Fig 4:**
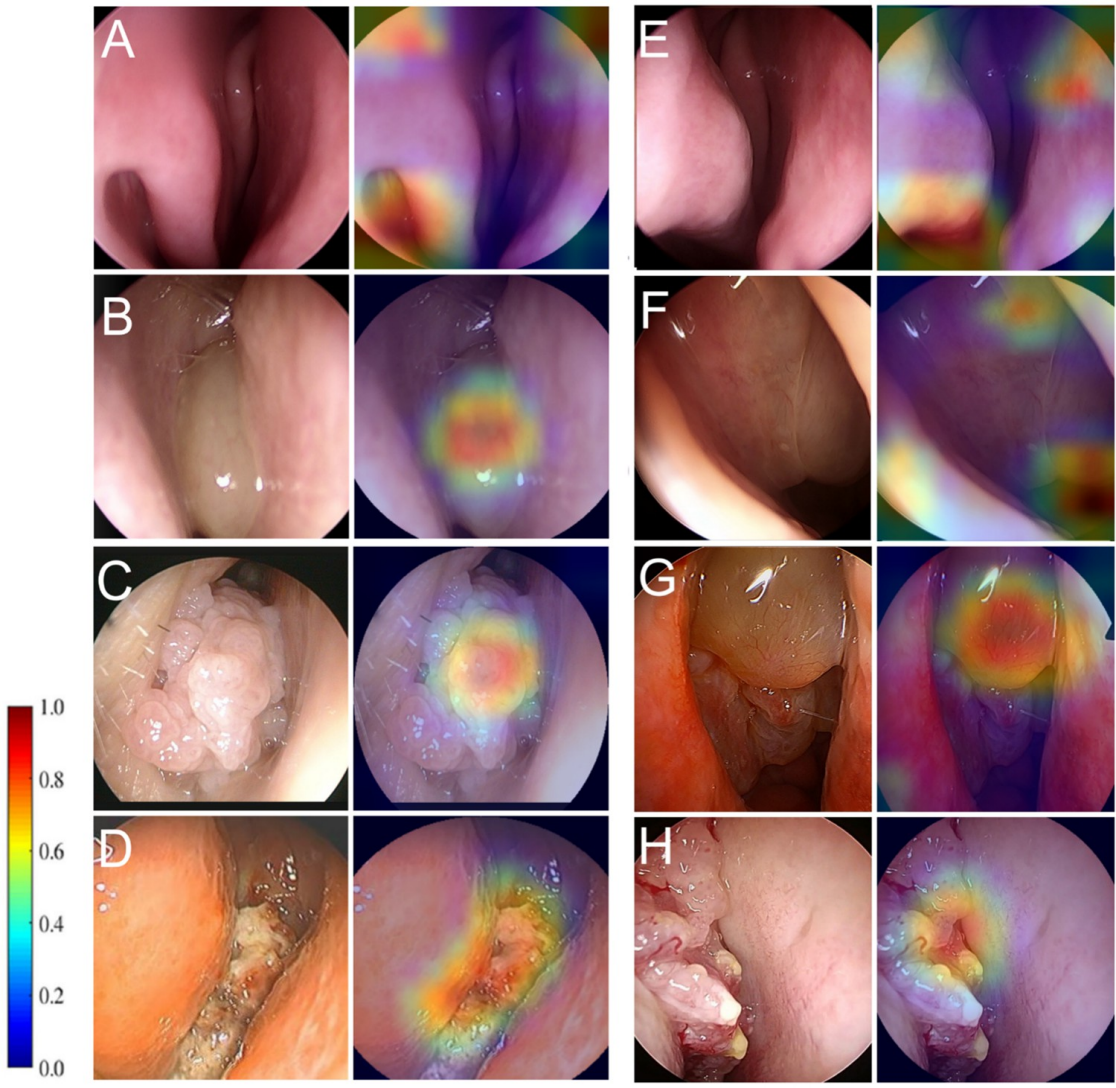
Visualized attention maps obtained by the proposed Xception deep learning model. (A)–(D) Attention maps for the (A) normal, (B) NP, (C) benign tumor (IP) and (D) malignant mass (SCC) classes. (E)–(H) Representative attention maps that were misclassified. (E) Normal image incorrectly classified as malignant mass, in which the inferior turbinate was confused with a tumor. (F) NP image incorrectly classified as benign tumor. (G) Benign tumor (IP) image incorrectly classified as an NP. (H) Malignant tumor (SCC) image incorrectly classified as a benign tumor. In the attention maps displayed as heat maps, warmer colors indicate higher saliency, that is, a higher contribution to the classification decision.

TensorBoard (https://tensorflow.org/tensorboard), a machine learning developer tool, was used to monitor the performance of the model during training. To visualize the test performance using metrics such as the receiver operating characteristic (ROC) curve and confusion matrix, a visualization library for Python (4.0.1) and Matplotlib (3.4.2) was used. Performance metrics, such as the F1-score and the area under the ROC curve (AUC) value, were generated using Python data analysis libraries, Keras, and scikit-learn (1.0.2). The preprocessed images were input into the proposed network, and objective metrics, including sensitivity, specificity, F1-score, overall accuracy, mean average precision (mAP), and AUC, were used to validate the performance of the proposed classification model. S1 File includes detailed information on these metrics. The gradient-weighted class activation mapping (Grad-CAM) method was combined with a localized attention map to determine how the proposed CNN model made classification decisions on for the input images (Fig 4).

### Comparison between the machine learning model and CVA

The performances of manual assessment and trained Xception machine learning model were compared on the four-class classification task. Eighteen otolaryngologists classified the raw 400 images in the test set into four classes based on CVA. Among the clinicians, six otolaryngologists were board-certified rhinologists with more than 5 years of experience in nasal endoscopy, another six otolaryngologists were senior residents with 3–4 years of experience, and the remaining six otolaryngologists were junior residents with 1–2 years of experience from a single institution. Assessments were conducted according to the appearance of the nasal endoscopic images without time constraints or any information on patient history. In addition to the four-class categorization, diagnostic performance was assessed for binary predictions on urgent (malignant lesion) versus non-urgent (non-malignant lesion) subjects based on sensitivity, specificity, F1-score, overall accuracy, and confusion matrix.

### Statistical analysis

Student’s *t*-test was conducted to compare the accuracy between the clinician groups. Sensitivity and specificity were analyzed using ROC curves and dichotomized tables. F1-score was calculated using the following formula: TP/(TP+0.5(FP+FN)), where TP represents the number of true positives, and FP and FN are the numbers of false positives and false negatives, respectively. The overall accuracy represents the ratio of the number of correctly classified images to the total number of test images. ROC curves and AUC were calculated using R Studio (RStudio Team, 2021) with R version 4.1.1 and the pROC package. The confusion matrices of the four-class classification task was evaluated to compare the diagnostic accuracy for each pathological class and count the correct and incorrect predictions in each class. Statistical analyses were performed using the SPSS version 20 (SPSS Inc., Chicago, IL), pandas (1.3.5), scikit-learn (1.0.2), NumPy (1.20.3), Matplotlib (3.4.2), and OpenCV (4.0.1) Python packages. Statistical significance was set at < 0.05. The model structures were developed on a Xeon Gold 6248 (3.0 GHZ/24-core/205 W, 256 GB RAM) central processing unit with NVIDIA Tesla V100 graphic process units (32 GB).

## Results

### Diagnostic performance of the CNN models

**Fig 5:**
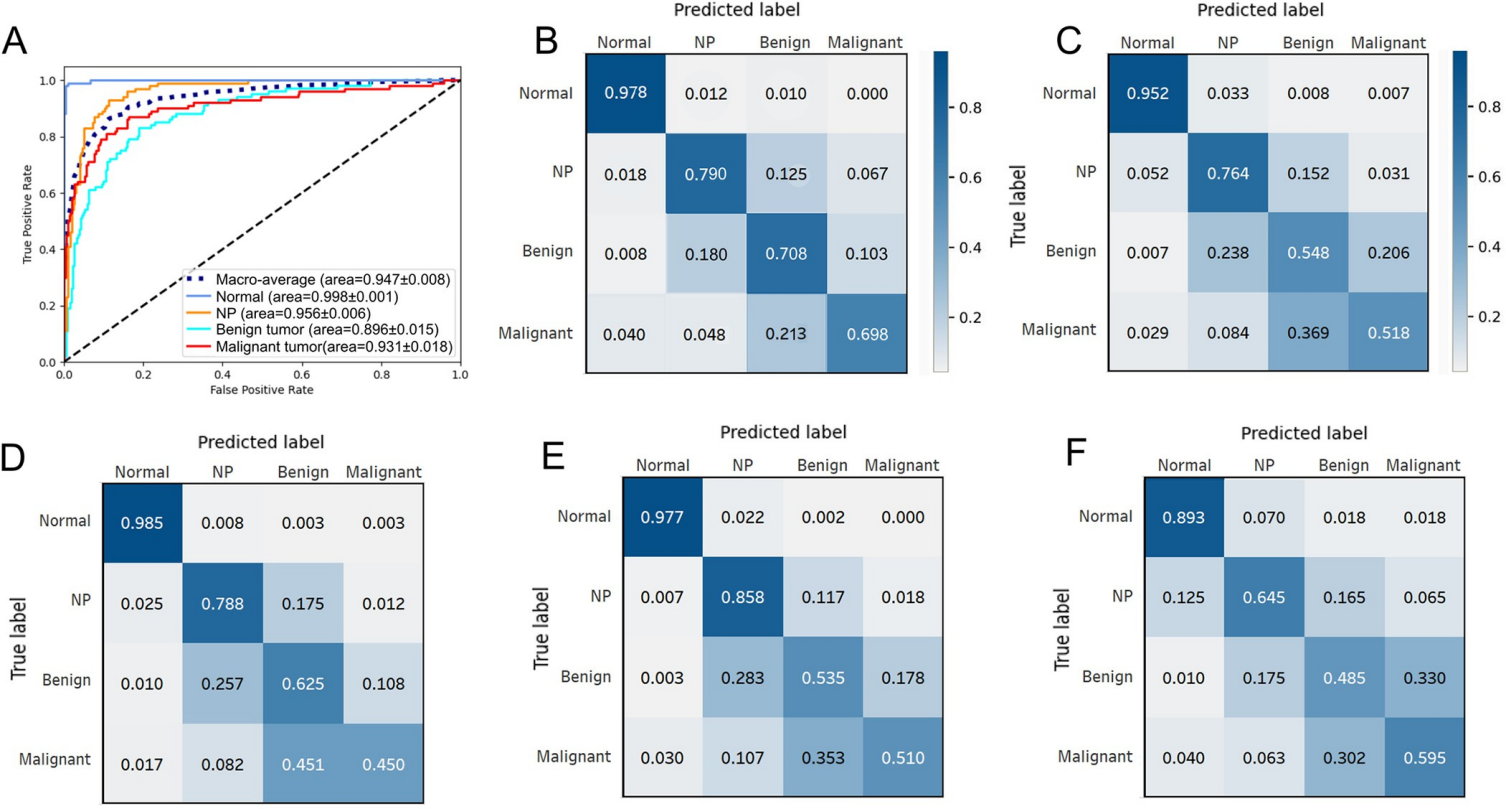
Performance of the Xception model and manual clinician visual assessment. Receiver operating characteristic (ROC) curves on the test set (A) and confusion matrix (B) of the Xception model. (C)–(F) Confusion matrices of the performance of clinician visual assessments by humans. (C) Average of all participants, including six rhinologists and twelve residents. (D) Average of six rhinologists. (E) Average of six senior residents. (F) Average of junior residents.

**Fig 6:**
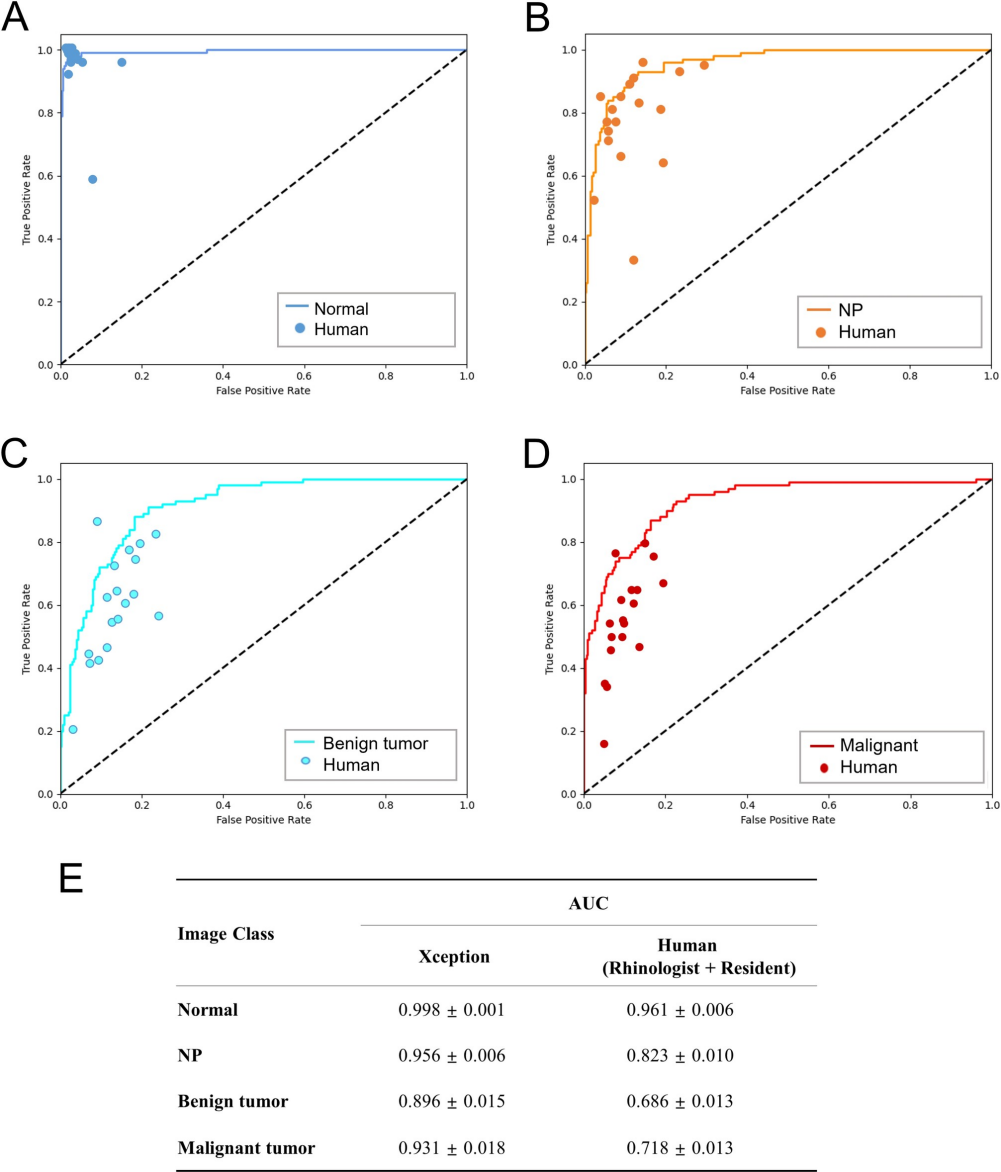
Comparison of the diagnostic performance for each class of the CNN model and eighteen otolaryngologists. ROC curves for normal (A), NP (B), benign tumor (C), malignant tumor (D) classes. AUC for the test set (E).

**Fig 7:**
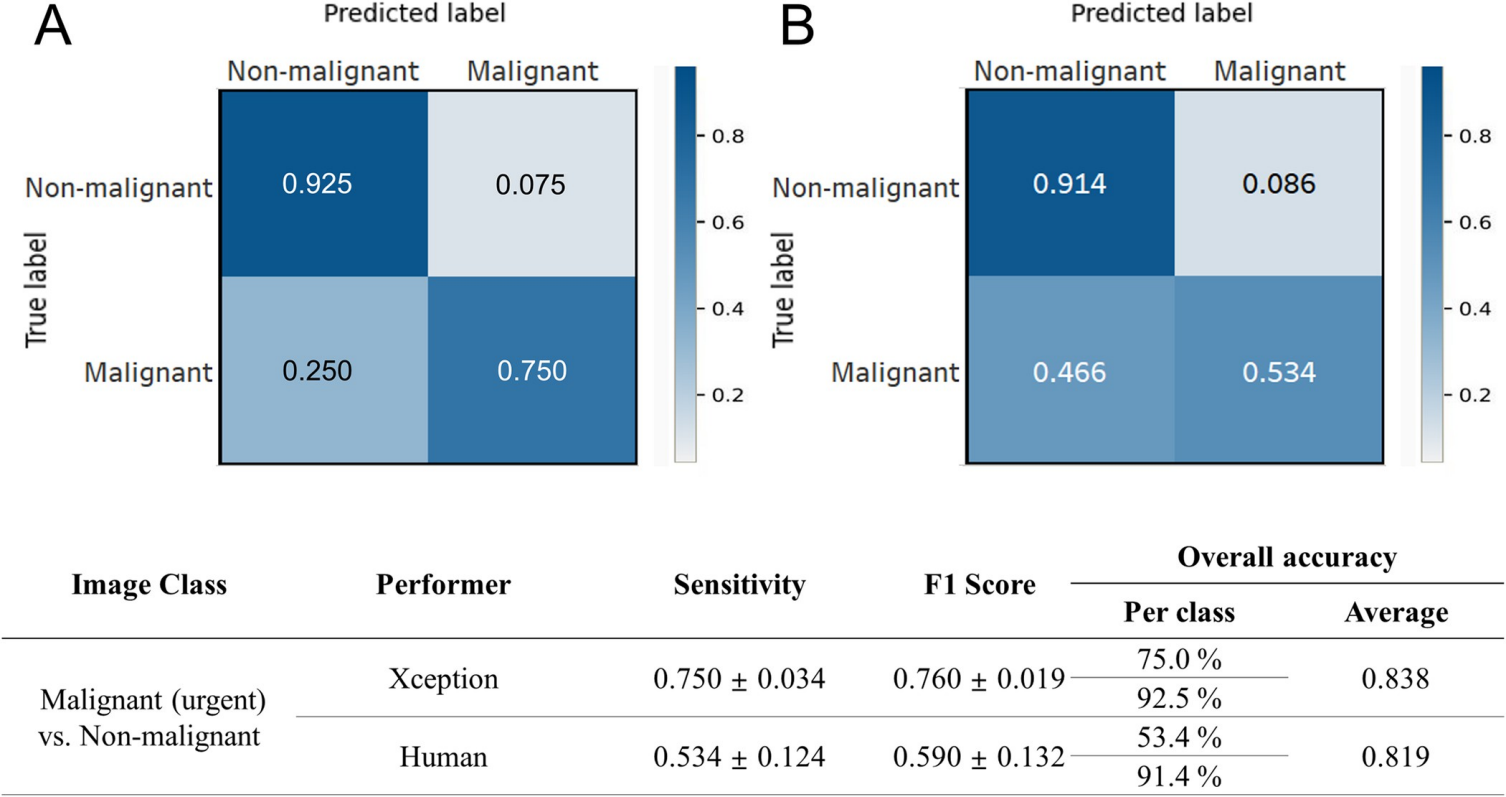
Urgent versus nonurgent binary classification performance. Results for the deep learning model (A) and human (B).

The details of the network setup, including the hyperparameter settings and weight values, are provided in S2 File, S2 and S3 Tables. A grid search method was used to identify the optimal set of parameter values for our networks, and a learning rate of 1e-04 and a batch size of 20 were selected. The models were run with each hyperparameter setting three times, and the best-performing model obtained accuracies of 0.927 ± 0.004 and 0.996 ± 0.001 (S1 File and S1 Table) on the validation and training sets, respectively. The validation loss for each machine learning model during training was assessed on the validation dataset (Fig 3). NasNetLarge obtained the lowest validation loss of 0.1018. InceptionResNet, Xception, ResNet152V2, and VGG19 obtained validation losses of 0.1251, 0.1303, 0.2572, and 0.3841, respectively. Subsequently, the performance of each CNN model with their optimal hyperparameter settings was evaluated on the test set (Table 1). The performance of each models was compared, and Xception was used as the backbone network in the final CNN model because it obtained the highest accuracy. The final classification performance results are listed in Table 2. The proposed model achieved an overall accuracy of 0.792 with the following class accuracies: normal = 0.978 ± 0.016, NP = 0.790 ± 0.016, benign = 0.708 ± 0.100, and malignant = 0.698 ± 0.116. The mean AUC was 0.947 ± 0.008, which indicates high performance in distinguishing between each class. Furthermore, the computed confusion matrix, ROC curves, and AUC values on the test set revealed the accuracy for normal class images was the highest, and the accuracy for malignant class images was the lowest (Figs 5A, 5B and 6). The confusion matrix reveals that the rate of malignant tumors misclassified as benign tumors (0.213) was higher than in any other case (Fig 5B). Furthermore, the lower AUC values for the benign and malignant classes were compared with the AUC results for the normal and NP classes (Fig 6E). The performance of binary prediction (urgent versus non-urgent cases) was evaluated. The proposed model achieved a sensitivity of 0.750 ± 0.034, F1-score of 0.760 ± 0.019, and average overall accuracy of 0.838. The urgent versus non-urgent performance is displayed as a confusion matrix in Fig 7A.

**Table 1:** Results of predictive performance indicators for the deep learning models.

Model	Sensitivity	Specificity	Precision	F1-Score	Accuracy	AUROC	mAP
**VGG19**	0.92	0.75	0.76	0.74	0.75	0.9	0.84
**ResNet152V2**	0.92	0.76	0.76	0.75	0.76	0.9	0.82
**InceptionResNetV2**	**0.94**	**0.82**	0.82	**0.82**	**0.82**	0.94	0.89
**N**as**NetLarge**	0.93	0.78	0.78	0.78	0.78	0.93	0.86
**Xception**	**0.94**	**0.82**	**0.84**	0.81	**0.82**	**0.96**	**0.90**

* Definitions of these metrics are given in S1 File.

**Table 2:** Comparisons of four-class image classification performance of human clinicians and the CNN model.

					Overall Accuracy	
Performer	Image Class	Sensitivity	Specificity	F1 Score	Per class	Average
**Rhinologist**	**Normal**	0.985 ± 0.015	0.983 ± 0.013	0.968 ± 0.025	98.5%	0.712
	**NP**	0.788 ± 0.149	0.885 ± 0.088	0.739 ± 0.081	78.8%	
	**Benign**	0.626 ± 0.136	0.790 ± 0.07	0.555 ± 0.107	62.6%	
	**Malignant**	0.450 ± 0.193	0.959 ± 0.030	0.550 ± 0.171	45.0%	
**Senior Resident**	**Normal**	0.977 ± 0.034	0.987 ± 0.005	0.968 ± 0.018	97.7%	0.710
	**NP**	0.858 ± 0.078	0.863 ± 0.091	0.762 ± 0.057	85.8%	
	**Benign**	0.535 ± 0.226	0.843 ± 0.071	0.509 ± 0.148	53.5%	
	**Malignant**	0.510 ± 0.106	0.934 ± 0.038	0.593 ± 0.070	51.0%	
**Junior Resident**	**Normal**	0.893 ± 0.169	0.942 ± 0.057	0.862 ± 0.014	89.3%	0.655
	**NP**	0.645 ± 0.176	0.897 ± 0.054	0.656 ± 0.159	64.5%	
	**Benign**	0.485 ± 0.137	0.838 ± 0.042	0.485 ± 0.072	48.5%	
	**Malignant**	0.595 ± 0.134	0.862 ± 0.062	0.589 ± 0.070	59.5%	
**All (Rhinologist + Resident)**	**Normal**	0.952 ± 0.103	0.970 ± 0.038	0.933 ± 0.093	95.2%	0.696
	**NP**	0.764 ± 0.160	0.881 ± 0.076	0.719 ± 0.112	76.4%	
	**Benign**	0.549 ± 0.172	0.823 ± 0.063	0.516 ± 0.111	54.9%	
	**Malignant**	0.518 ± 0.153	0.919 ± 0.060	0.577 ± 0.109	51.8%	
**Xception**	**Normal**	0.978 ± 0.016	0.978 ± 0.012	0.957 ± 0.016	97.8%	0.794
	**NP**	0.790 ± 0.016	0.920 ± 0.049	0.773 ± 0.051	79.0%	
	**Benign**	0.708 ± 0.100	0.886 ± 0.051	0.688 ± 0.025	70.8%	
	**Malignant**	0.698 ± 0.116	0.941 ± 0.035	0.742 ± 0.057	69.8%	

### Attention maps

Fig 4 visualizes how the Xception model classified the images by weighting all pixels according to their importance using Grad-CAM attention maps. Fig 4A–4D present examples of accurately classified images that indicate key lesions or structures that contribute to decision-making. Some representative misclassified images in Fig 4E–4H provide insight into why the CNN model made the wrong decisions.

### Comparison of the CNN model with manual assessment

A comparison of the four-group classification performance between the proposed model and that of manual assessment by clinicians is presented in Table 2. In both the CNN model and manual assessment, the accuracy was highest in the order of normal, NP, benign tumor, and malignant tumor. The CNN model achieved a higher overall accuracy than human clinicians and rhinologist expert subgroups (0.794 vs. 0.696 and 0.794 vs. 0.712, respectively). The accuracies for normal and NP classification of the CNN model was comparable to those of manual assessment (0.978 ± 0.016 vs. 0.952 ± 0.103; p = 0.792, 0.790 ± 0.016 vs. 0.764 ± 0.160; p = 0.716, respectively), and the proposed model outperformed manual assessment in terms of benign and malignant classification accuracies (0.708 ± 0.100 vs. 0.549 ± 0.172; p = 0.019, 0.698 ± 0.116 vs. 0.518 ± 0.153; p = 0.012, respectively). Furthermore, the CNN model exhibited F1-scores for benign and malignant classes that were higher than those of CVA (0.688 ± 0.025 vs. 0.516 ± 0.111; p < 0.001, 0.742 ± 0.057 vs. 0.577 ± 0.109; p < 0.001, respectively). The ROC curves demonstrate that the proposed model outperformed manual assessments in classifying nasal cavity mass images and exhibited considerably higher AUC values than clinicians in benign and malignant tumor classification (0.896 ± 0.015 vs. 0.686 ± 0.013; p < 0.001, 0.931 ± 0.018 vs. 0.718 ± 0.013; p < 0.001, respectively) (Fig 6). We compared the performance of the proposed CNN models with that of human clinicians in performing urgent versus non-urgent binary predictions. The results are displayed in Fig 7, in which the CNN model achieved significantly higher sensitivity and F1-scores than clinicians in urgent (malignant tumor) classification (0.750 ± 0.034 vs. 0.534 ± 0.124; p < 0.001, 0.760 ± 0.019 vs. 0.590 ± 0.132; p < 0.001).

## Discussion

A CNN-based deep learning model was developed for the automatic detection and classification of tumorous conditions in NP, benign, and malignant tumors using nasal endoscopic images. The results revealed that CNN-based models can be trained to detect tumorous lesions in the nasal cavity as well as differentiate between benign and malignant lesions. The deep learning model trained on nasal endoscopic images achieved acceptable prediction performance in the diagnosis of nasal cavity lesions. Notably, the model for classifying benign and malignant tumors of the nasal cavity outperformed manual assessment.

Classifying nasal cavity mass lesions according to their histopathological features helps us understand their clinical presentation, treatment, clinical outcomes, and prognosis [18–22]. Nasal endoscopy is a safe and rapid method that is becoming increasingly popular in the clinical setting for otolaryngologists and has become the gold standard for nasal cavity examinations [23]. Pathological nasal cavity mass lesions generally have characteristic appearances, which can be easily diagnosed by visual assessment using endoscopy. For example, a typical NP is a semitransparent, pale gray, or pinkish lobular mucosal tissue with a smooth and glossy surface (Fig 4B) [21]. Similarly, IP is a pale, polypoid mass but has a more irregular vascularized surface, and a granular mulberry-like appearance (Fig 4C) [18]. However, the endoscopic visual assessment of nasal cavity lesions is subjective and requires considerable experience [17]. In this study, the diagnostic accuracy of CVA of human participants was investigated using endoscopic images without any prior knowledge of patient history, and the outcome was highly dependent on the pathologic class. The AUC results revealed inferior diagnostic performance for the benign tumor and malignant tumor classes on the test set. In particular, the overall accuracy of malignant tumors was approximately half that of the normal class. Notably, among participant groups, classification accuracy for malignant tumors was the highest in the junior resident group, despite the rhinology-specialist group achieving higher average overall accuracy. This phenomenon supports the notion [17] that clinicians encounter considerable difficulty in distinguishing tumorous lesions from normal and NP lesions, irrespective of the level of experience of the examiner. We compared the performance of the trained deep learning model with that of manual assessment on the binary prediction of urgent versus nonurgent classes. The results validated the effectiveness of the CNN model in detecting urgent (malignant) cases. The binary classification task is of clinical significance because the majority of malignant tumors require radical and immediate treatment with regular follow-up for the early detection of recurrence or metastases, and misdiagnosis may delay treatment for the problem, which can be fatal [20, 24]. In particular, as displayed by the confusion matrices of the performance results, the rate of misclassification of malignant tumors as benign tumors is higher than other cases for both the CNN model and manual assessment, which is understandable considering the similarity of the gross appearance of lesions (Fig 5B and 5C).

Deep learning models require a considerable amount of data for training. However, obtaining sufficient clinical data is difficult because sinonasal tumors are rare. We addressed this problem using a transfer learning method [25, 26] by employing a pretrained Xception model as an initial framework, which was fine-tuned on the target dataset. Xception, a CNN architecture based on a depth-wise separable convolution layer, is a modification of Inception, a high performing network [27]. Although Inception and Xception have similar features and numbers of parameter, Xception exhibits slightly superior performance on the ImageNet dataset [11] and significantly superior performance on the JFT dataset [28]. Of the five deep neural network architectures considered in our study, NasNetLarge achieved the lowest validation loss, but Xception, which achieved the best values in terms of most performance metrics on the test set, was used as the final prediction model. The superior results of NasNetLarge and Xception could be attributed to the difference in the image resolutions of the models. According to the required input dimensions of each model, the images were resized during preprocessing to 331 × 331 pixels for NasNetLarge and Xception and 224 × 224 pixels for VGG19, ResNet152V2, and InceptionResNetV2. Thambawita et al. [29] investigated various resolutions and their effects on CNNs. The results revealed increased performance with high image resolution. Xception outperformed NasNetLarge on the test set because the parameters are efficiently used in Xception, even though the NasNetLarge architecture has more parameter capacity than Xception (88.9M vs. 22.9M). Similarly, Yoon et al. [30] comprehensively analyzed 292 studies of COVID-19 diagnosis in which artificial intelligence was used on chest X-ray images, and it was found that the best results were achieved using the algorithm based on MobileNet and the Xception architecture. In most of the studies mentioned above [30], two or three classes, and 1,001–10,000 images were used as the database, and the average accuracy, AUC, sensitivity, and specificity were 95.2%, 94.01%, 93.5%, and 93.92%, respectively. Similarly, combined with the transfer learning technique, CNNs have been more widely used in medical imaging analysis for various clinical applications [15, 31–39]. However, the feasibility of this network in nasal endoscopy has not yet been established because of the heterogeneity of nasal cavity lesions and structural complexity of the nasal cavity. To the best of our knowledge, this study is the first to elucidate the applicability of deep learning algorithms in detecting and classifying nasal endoscopic images as normal, NP, benign, or malignant tumors. The results indicate that the trained CNN model can be used for diagnosing nasal cavity tumors. In the future, this algorithm-based diagnostic tool can be used for early diagnosis and timely treatment in clinical settings, thereby reducing the morbidity and mortality of nasal cavity mass lesions, especially in malignant cases.

Studies have revealed that CNNs can achieve comparable or higher performance than manual assessment. These studies have focused on their ability to classify malignant lesions in microscopic or endoscopic images in various clinical fields. Heo et al. [40] developed a deep learning model to detect tongue cancer based on oral endoscopic images and achieved an accuracy of 84.7% using the DenseNet169 model. The accuracy of the human oncology specialist was higher than that of the model (92%), whereas that of the general physician was lower (75.9%). In a study by Yang et al. [41], a classifier based on Inception V3 proposed for laryngeal neoplasms using laryngoscopy images exhibited excellent performance in distinguishing malignancies between the algorithm model and physicians (90% vs. 54%). In another similar study [42], the accuracy of a CNN model in detecting laryngeal cancer was 0.773, which was comparable to that of human experts with 10–20 years of work experience and exceeded that of experts with less than 10 years of experience. Li et al. [43] used an endoscopic image-based deep learning model based on the Inception architecture to discriminate nasopharyngeal malignancies, which outperformed manual assessment with an accuracy of 88.0% versus 80.5%.

With the new revolution in artificial intelligence-based medical diagnosis, neural networks have been used to analyze images based on instances observed during training and subsequently identify and extract relevant information from a given image. Therefore, a large training dataset, such as one with at least 1000 training examples for each class [30], is required to obtain validated results. Implementing a deep learning method with unevenly distributed data for each class can result in biased classification due to the overfitting of certain classes with large samples. However, the collection of raw data can be time-consuming and expensive. Collecting data for tumor lesions, especially malignant tumors, in this study was also highly challenging because of the rarity of their occurrences, whereas sufficient images were available for normal and NP cases. To address this data imbalance, various class weights were applied according to the number of samples per class (S2 File). Augmentations, including rotation, zooming, and two types of sharpening filters, were applied to the collected dataset.

Furthermore, we investigated the recognition of the model for diagnosis by analyzing the Grad-CAM attention map. The trained model could comprehensively determine the smoothness, roughness, and color characteristics of the tumor surface. Notably, when detecting normal nasal cavity images, the inferior turbinate and septum, but not the middle turbinate, were recognized. However, misclassified examples provide insight into how the models made erroneous decisions. For example, a normal nasal cavity structure, such as an inferior/middle turbinate, and smooth-surface malignant tumors were misclassified as benign tumors. Decision-making accuracy was low for SCC dysplasia due to IP or the concomitant presence of inflammatory polyps.

This study has some inherent limitations. First, the collaboration with many institutions to obtain sufficient data was challenging because of the rarity of sinonasal tumors. The detection efficiency for benign and malignant tumors could therefore be lower in practice. Although we attempted to avoid overfitting using advanced methods such as transfer learning and augmentation, the reduced diversity and small number of images mean that the model may not be as effective as those of other studies trained on rich datasets or CT/MRI images. Further large-scale multicenter studies with additional lesion types and a larger number of malignant images in particular should be performed to achieve diagnostic accuracy in real clinical settings. Second, the number of collected images, image format, and endoscopic device varied by institution, which suggests that our data may be biased. Therefore, caution should be exercised when interpreting our results. In particular, the image quality and lighting conditions during endoscopy, which are contingent on center-specific equipment, vary widely and influence the feature clarity and contrast, complicating model training. For robust deep learning models, generalizability should be evaluated with sufficient training and validation samples as well as independent testing using internal and external multi-institutional data. Third, the explanatory power of our model is limited because it is a classification method rather than an object detection method, as used in other endoscopy dataset studies. Therefore, it will be necessary to consider an object detection model in the future, and we plan to perform research using models such as a Region CNN or You Only Look Once (YOLO). Fourth, compared with other medical imaging data, nasal endoscopic data present unique challenges when used to train deep learning models. Unlike endoscopic images from procedures such as laryngoscopy, colonoscopy, or gastroenteroscopy, nasal endoscopic images usually have a high degree of dimensionality due to the complex anatomic structure of the nasal cavity. This can make the data more challenging to work with than lower-dimensional data, such as ophthalmic images, chest, or paranasal sinus radiographs. In addition, a nasal endoscopy image shows intranasal lesions in a two-dimensional cross-section, which cannot completely represent the mass in the nasal cavity with complex structures and anatomical locations. To evaluate diagnostic CVA performance in future studies, we plan to consider endoscopic video recordings, which are time series data, using an approach similar to that of Xiao et al. [44]. In this manner, the size and diversity of tumor datasets can be increased.

Our study introduces and evaluates the application of deep learning to the less explored field of nasal endoscopic imaging, thus laying the groundwork for future research. The adaptation of deep-learning techniques to classify a wide range of nasal endoscopic findings and comparable performance of these techniques with medical professionals demonstrate the feasibility and potential of AI to support clinical decision making. Considering practical applications, our results are poised to inspire the development of AI-powered tools such as automated diagnostic systems for endoscopic images or real-time AI-assisted nasal endoscopy, akin to advancements in gastroenteroscopy to assist clinicians in disease screening during endoscopy procedures [45].

## Conclusion

An endoscopic image-based deep learning model was developed using Xception to achieve high diagnostic accuracy in the automated detection of four classes of nasal cavity mass lesions. The trained model showed superior classification performance for the benign and malignant tumor classes compared with human clinicians. In the future, improved algorithms with acceptable diagnostic accuracy could be used for the early screening of nasal cavity lesions and to assist clinicians, thereby increasing work efficiency.

## Supporting information

S1 File Performance evaluation metrics. (DOCX)

S2 File Network training set up. (DOCX)

S1 Table Performance on the entire hyperparameter set. (DOCX)

S2 Table Grid of hyperparameters. (DOCX)

S3 Table Total number of trainable and non-trainable parameters of the CNN model. (DOCX)
