# Supplementary material for: Deep learning algorithm for the automated detection and classification of nasal cavity mass in nasal endoscopic images
Source: PLoS One. 2024 Mar 13;19(3):e0297536. doi: 10.1371/journal.pone.0297536 (PMC10936791; doi:10.1371/journal.pone.0297536)
Supplement: S1 File — (DOCX) [file pone.0297536.s001.docx]

**S1 File. Performance Evaluation Metrics**

**Accuracy** represents the ratio of correctly classified images and is calculated as follows:

$$Accuracy=\frac{Total Number of Correctly Classified Images}{Total Number of Considered Images}$$

The confidence interval (CI) was computed as follows:

$CI=\mu\pm t\frac{s}{\sqrt{n}}$,

where$\mu$ is the sample mean, $s$ is the sample standard deviation, $n$ is the sample size, and $t$ is the t-statistic, depending on a 95% CI. Because of the low number of human experts (*n* = 18) and Xception model (*n* = 6), the confidence intervals (CIs) were calculated using a t-statistic instead of a z-score, as suggested in [J. C. F. de Winter. ‘Using the Student’s t-test with extremely small sample sizes.’ *Practical Assessment, Research, and Evaluation*, Vol. 18, 2013. <https://doi.org/10.7275/e4r6-dj05>].

For the ROC, the true-positive rate (TPR) and false-positive rate (FPR) were obtained as follows:

$$TPR=\frac{TP}{TP+FN}$$

$$FPR=\frac{FP}{FP+TN}$$

where TP represents the number of true positives and TN, FP, and FN are the numbers of true negatives, false positives, and false negatives, respectively. The **AUC (**or **AUROC)** is the area under the ROC curve. The ROC for multilabel classification was obtained by binarizing the output into two cases: one for the target class and the other for the rest of the classes. The ROC curves and AUCs were calculated using Python library scikit. The ROC curve function was used to compute the ROC curves. Because this function returns only the necessary FPRs (*y*-axis values) corresponding to certain TPRs (*x*-axis values), the returned values differ in length in the folds we used. Therefore, to average the ROC curves, the following steps were used. First, the TPRs (x-axis values) were uniformly spaced at 1/101 intervals. The corresponding FPRs (y-axis values) were then obtained by linearly interpolating the initially returned rates. Subsequently, the ROC curves were averaged over the ten folds.

**Sensitivity** represents the TPR, which is the probability of a positive test result conditioned on the individual truly being positive. **Specificity**, the TNR, is the probability of a negative test result conditioned on the individual truly being negative. **Precision** reflects how reliable the model is at classifying samples as positive. These metrics were calculated as follows:

$$Sensitivity (TPR)=\frac{TP}{TP+FN}$$

$$Specificity (TNR)=\frac{TN}{FP+TN}$$

$$Precision=\frac{TP}{TP+FP}$$

The **F1 score** was calculated by binarizing the confusion matrices on the test set for the models and human experts and then applying the following formula:

$$F1=\frac{TP}{TP+0.5(FP+FN)}$$

The **average precision (AP)** was computed using the scikit learn function, called average_precision_score, as follows:

$$AP= \sum_{n} \left( R_{n}-R_{n-1} \right)P_{n}$$

where $R_{n}$ and $P_{n}$ are recall and precision at the *n*th threshold, respectively. This implementation is not interpolated and differs from computing the area under the precision–recall curve with the trapezoidal rule in which linear interpolation is used, which can be too optimistic.

All values reported were rounded to the nearest hundredth or thousandth; that is, five and higher values were rounded up at the last digit.
