# Supplementary material for: Deep learning algorithm for the automated detection and classification of nasal cavity mass in nasal endoscopic images
Source: PLoS One. 2024 Mar 13;19(3):e0297536. doi: 10.1371/journal.pone.0297536 (PMC10936791; doi:10.1371/journal.pone.0297536)
Supplement: S2 File — (DOCX) [file pone.0297536.s002.docx]

**S2 File. Network Training Setup**

An epoch is typically defined as a complete iteration through a training dataset. We chose to train our model using Adam as defined in the following equations:

$m_{t}=\beta_{1}m_{t-1}+\left( 1-\beta_{1} \right)g_{t}$ (1)

$v_{t}=\beta_{2}v_{t-1}+(1-\beta_{2})g_{t}^{2}$ (2)

${m ̂}_{t}= \frac{m_{t}}{1-\beta_{1}^{t}}$ (3)

$\hat{v}_{t}=\frac{v_{t}}{1-\beta_{2}^{t}}$ (4)

$\theta_{t+1}= \theta_{t}-\frac{\eta}{\sqrt{\hat{v}_{t}}+\epsilon}\hat{m}_{t}$ (5)

where $\theta$ is a parameter of our neural network, $m_{t}$ is the momentum, $\eta$ is the learning rate, $v_{t}$ is exponentially decaying average of the past squared gradients, $\beta_{1}$ is the exponential decay rate for the first moment estimates, $\beta_{2}$ is the exponential decay rate for the second moment estimates, and $\epsilon$ is a small constant used to ensure numerical stability [1]. To fairly compare the different hyperparameter sets, all the parameter sets were compared for 10 epochs because most of the settings exhibited reasonable convergence at this stage. The same loss function was applied in all the models that were used and trained over ImageNet [2]; that is, the cross-entropy loss was set. Thus, the output of the final layer should be transformed into a probability value, which was achieved by passing the outputs of our model through a softmax function. Thus, loss is defined as follows:

$$L_{i}\left( \theta\right)=-log\left( \frac{e^{f_{\theta}(x_{i})^{(k)}}}{\Sigma_{m}e^{f_{\theta}(x_{i})^{(m)}}} \right)$$

where $f_{\theta}(x_{i})^{(k)}$ is the $i^{th}$component of the output of our neural network with parameter $\theta$ on image $x_{i}$ classified as diagnosis $k$. Here, $m$ represents the number of classes, that is, $m=2 or 4$.

To avoid overfitting, different class weights were applied depending on the number of images per class. The class weights are defined as follows:

$$W_{i}=\frac{1}{\mathrm{NumClass}_{i}}\frac{Total Img}{Total Class}$$

where *i* indicates the class, *Num Class* is the number of images corresponding to class *i*, *Total Img* is the total number of images in the training dataset, and *Total Class* is the number of classes.

Various values were considered for the following parameters: number of pretrained batch sizes, weight initialization type, and learning rate ($\eta$ in Equation 1). A grid search was performed over the following possible values presented in **S2 Table**: batch sizes of 10, 15, 20, and 30 and learning rates of 0.01, 0.001, 0.0001, and 0.00001.

Regarding the software and hardware components used for our research work, we performed image preprocessing and CNN training using the following tools and libraries:

- Python version 3.8.11
- TensorBoard version 2.9.0
- TensorFlow version 2.4.1
- Keras version 2.4.3
- scikit-learn version 1.0.2
- NumPy version 1.20.3
- OpenCV version 4.0.1

**References**

1. Ruder, S., *An overview of gradient descent optimization algorithms*. 2016.
2. Deng, J., et al. *ImageNet: A large-scale hierarchical image database*. 2009.
