# Supplementary material for: Deep learning algorithm for the automated detection and classification of nasal cavity mass in nasal endoscopic images
Source: PLoS One. 2024 Mar 13;19(3):e0297536. doi: 10.1371/journal.pone.0297536 (PMC10936791; doi:10.1371/journal.pone.0297536)
Supplement: S1 Table — (DOCX) [file pone.0297536.s003.docx]

**S1 Table. Performance on the entire hyperparameter set**

Here, $\eta$ represents the learning rate, and the values are sorted based on validation accuracy

| model | fit | | score | | batch size | η | rank | Validation | | Train | |
| --- | --- | --- | --- | --- | --- | --- | --- | --- | --- | --- | --- |
|  | mean time | STD time | mean time | STD time |  |  |  | mean | STD | mean | STD |
| Xception | 1561.535 | 1.722 | 25.344 | 0.069 | 20 | 0.0001 | 1 | 0.927 | 0.004 | 0.996 | 0.001 |
| Xception | 1673.529 | 5.913 | 28.111 | 0.238 | 10 | 0.0001 | 2 | 0.915 | 0.009 | 0.991 | 0.004 |
| Xception | 1603.944 | 3.450 | 26.146 | 0.038 | 15 | 0.0001 | 3 | 0.914 | 0.010 | 0.992 | 0.004 |
| Xception | 1512.069 | 1.259 | 24.332 | 0.011 | 30 | 0.0001 | 4 | 0.906 | 0.006 | 0.993 | 0.002 |
| Xception | 1737.036 | 93.274 | 45.325 | 23.979 | 10 | 1.00E-05 | 5 | 0.874 | 0.005 | 0.992 | 0.000 |
| Xception | 1709.623 | 157.180 | 26.259 | 0.075 | 15 | 1.00E-05 | 6 | 0.869 | 0.003 | 0.991 | 0.001 |
| Xception | 1701.762 | 197.905 | 25.421 | 0.022 | 20 | 1.00E-05 | 7 | 0.857 | 0.004 | 0.987 | 0.002 |
| Xception | 1578.638 | 97.276 | 24.396 | 0.048 | 30 | 1.00E-05 | 8 | 0.843 | 0.006 | 0.974 | 0.002 |
| Xception | 1514.505 | 2.134 | 24.397 | 0.028 | 30 | 0.001 | 9 | 0.837 | 0.019 | 0.925 | 0.018 |
| Xception | 1610.043 | 1.801 | 25.977 | 0.176 | 15 | 0.001 | 10 | 0.818 | 0.037 | 0.907 | 0.039 |
| Xception | 1559.923 | 1.179 | 25.413 | 0.128 | 20 | 0.001 | 11 | 0.798 | 0.022 | 0.886 | 0.028 |
| Xception | 1672.015 | 0.989 | 28.397 | 0.058 | 10 | 0.001 | 12 | 0.773 | 0.073 | 0.851 | 0.087 |
| Xception | 1680.876 | 5.845 | 28.579 | 0.178 | 10 | 0.01 | 13 | 0.665 | 0.028 | 0.676 | 0.029 |
| Xception | 1710.333 | 131.151 | 26.139 | 0.040 | 15 | 0.01 | 14 | 0.633 | 0.029 | 0.640 | 0.025 |
| Xception | 1513.458 | 2.801 | 24.326 | 0.086 | 30 | 0.01 | 15 | 0.580 | 0.079 | 0.590 | 0.079 |
| Xception | 1561.898 | 5.060 | 25.357 | 0.029 | 20 | 0.01 | 16 | 0.576 | 0.078 | 0.579 | 0.081 |
| Inception ResNet V2 | 585.117 | 1.068 | 21.241 | 0.142 | 30 | 0.0001 | 1 | 0.916 | 0.011 | 0.985 | 0.006 |
| Inception ResNet V2 | 774.490 | 1.430 | 27.471 | 0.237 | 15 | 0.0001 | 2 | 0.909 | 0.005 | 0.980 | 0.004 |
| Inception ResNet V2 | 664.567 | 4.644 | 23.854 | 0.475 | 20 | 0.0001 | 3 | 0.901 | 0.031 | 0.974 | 0.023 |
| Inception ResNet V2 | 950.933 | 3.619 | 35.282 | 0.272 | 10 | 0.0001 | 4 | 0.899 | 0.018 | 0.975 | 0.012 |
| Inception ResNet V2 | 1087.464 | 197.124 | 68.772 | 47.037 | 10 | 1.00E-05 | 5 | 0.880 | 0.015 | 0.985 | 0.007 |
| Inception ResNet V2 | 1025.672 | 351.348 | 27.433 | 0.410 | 15 | 1.00E-05 | 6 | 0.872 | 0.002 | 0.990 | 0.003 |
| Inception ResNet V2 | 956.480 | 420.197 | 24.038 | 0.186 | 20 | 1.00E-05 | 7 | 0.864 | 0.005 | 0.988 | 0.003 |
| Inception ResNet V2 | 755.090 | 238.537 | 21.454 | 0.262 | 30 | 1.00E-05 | 8 | 0.847 | 0.010 | 0.992 | 0.000 |
| Inception ResNet V2 | 666.986 | 1.918 | 24.100 | 0.090 | 20 | 0.001 | 9 | 0.825 | 0.024 | 0.905 | 0.026 |
| Inception ResNet V2 | 584.591 | 0.381 | 21.103 | 0.160 | 30 | 0.001 | 10 | 0.779 | 0.014 | 0.858 | 0.022 |
| Inception ResNet V2 | 772.456 | 0.792 | 27.242 | 0.188 | 15 | 0.001 | 11 | 0.750 | 0.026 | 0.814 | 0.035 |
| Inception ResNet V2 | 951.074 | 0.755 | 35.333 | 0.302 | 10 | 0.001 | 12 | 0.719 | 0.055 | 0.775 | 0.066 |
| Inception ResNet V2 | 669.613 | 1.541 | 24.198 | 0.128 | 20 | 0.01 | 13 | 0.587 | 0.054 | 0.598 | 0.052 |
| Inception ResNet V2 | 586.634 | 0.943 | 21.157 | 0.147 | 30 | 0.01 | 14 | 0.584 | 0.039 | 0.588 | 0.048 |
| Inception ResNet V2 | 773.861 | 4.085 | 27.361 | 0.128 | 15 | 0.01 | 15 | 0.527 | 0.027 | 0.531 | 0.024 |
| Inception ResNet V2 | 952.306 | 2.924 | 35.314 | 0.179 | 10 | 0.01 | 16 | 0.484 | 0.112 | 0.491 | 0.118 |
| NASNetLarge | 5419.342 | 137.368 | 110.837 | 23.131 | 10 | 1.00E-05 | 1 | 0.914 | 0.011 | 0.997 | 0.002 |
| NASNetLarge | 5272.165 | 266.409 | 84.597 | 0.797 | 15 | 1.00E-05 | 2 | 0.893 | 0.002 | 0.994 | 0.001 |
| NASNetLarge | 5343.512 | 5.498 | 94.005 | 0.403 | 10 | 0.0001 | 3 | 0.867 | 0.025 | 0.936 | 0.025 |
| NASNetLarge | 5122.415 | 306.021 | 80.718 | 0.410 | 20 | 1.00E-05 | 4 | 0.856 | 0.010 | 0.962 | 0.009 |
| NASNetLarge | 5090.219 | 2.241 | 85.754 | 0.216 | 15 | 0.0001 | 5 | 0.830 | 0.034 | 0.899 | 0.032 |
| NASNetLarge | 4904.986 | 2.438 | 80.815 | 0.171 | 20 | 0.0001 | 6 | 0.820 | 0.030 | 0.885 | 0.031 |
| NASNetLarge | 4741.925 | 1.478 | 74.624 | 0.073 | 30 | 0.0001 | 7 | 0.812 | 0.015 | 0.871 | 0.018 |
| NASNetLarge | 4849.428 | 156.912 | 74.577 | 0.443 | 30 | 1.00E-05 | 8 | 0.777 | 0.020 | 0.851 | 0.027 |
| NASNetLarge | 4743.222 | 5.735 | 74.514 | 0.258 | 30 | 0.001 | 9 | 0.365 | 0.006 | 0.365 | 0.004 |
| NASNetLarge | 4738.512 | 0.989 | 74.569 | 0.273 | 30 | 0.01 | 10 | 0.335 | 0.043 | 0.336 | 0.044 |
| NASNetLarge | 5338.072 | 2.085 | 93.873 | 0.283 | 10 | 0.01 | 11 | 0.327 | 0.051 | 0.332 | 0.051 |
| NASNetLarge | 4903.009 | 3.219 | 80.970 | 0.313 | 20 | 0.01 | 12 | 0.310 | 0.065 | 0.311 | 0.061 |
| NASNetLarge | 5087.273 | 4.733 | 85.491 | 0.223 | 15 | 0.01 | 13 | 0.309 | 0.045 | 0.302 | 0.043 |
| NASNetLarge | 5086.120 | 3.345 | 85.754 | 0.291 | 15 | 0.001 | 14 | 0.301 | 0.074 | 0.305 | 0.074 |
| NASNetLarge | 5340.229 | 3.252 | 94.234 | 0.563 | 10 | 0.001 | 15 | 0.281 | 0.058 | 0.285 | 0.063 |
| NASNetLarge | 4903.672 | 1.761 | 80.766 | 0.126 | 20 | 0.001 | 16 | 0.257 | 0.021 | 0.259 | 0.026 |
| VGG19 | 602.541 | 50.381 | 26.754 | 10.752 | 10 | 1.00E-05 | 1 | 0.835 | 0.014 | 0.976 | 0.006 |
| VGG19 | 591.591 | 130.576 | 16.690 | 0.123 | 20 | 1.00E-05 | 2 | 0.833 | 0.013 | 0.986 | 0.006 |
| VGG19 | 485.128 | 95.127 | 12.963 | 0.257 | 30 | 1.00E-05 | 3 | 0.825 | 0.005 | 0.990 | 0.002 |
| VGG19 | 564.837 | 147.351 | 14.402 | 0.026 | 15 | 1.00E-05 | 4 | 0.824 | 0.016 | 0.973 | 0.011 |
| VGG19 | 418.034 | 0.695 | 12.827 | 0.038 | 30 | 0.0001 | 5 | 0.700 | 0.011 | 0.714 | 0.021 |
| VGG19 | 499.603 | 0.764 | 16.476 | 0.040 | 20 | 0.0001 | 6 | 0.662 | 0.030 | 0.677 | 0.030 |
| VGG19 | 565.800 | 0.324 | 19.071 | 0.074 | 10 | 0.0001 | 7 | 0.510 | 0.163 | 0.511 | 0.170 |
| VGG19 | 460.275 | 0.048 | 14.668 | 0.310 | 15 | 0.0001 | 8 | 0.408 | 0.190 | 0.411 | 0.193 |
| VGG19 | 563.619 | 0.339 | 18.989 | 0.001 | 10 | 0.01 | 9 | 0.335 | 0.043 | 0.336 | 0.044 |
| VGG19 | 564.585 | 0.524 | 19.001 | 0.077 | 10 | 0.001 | 10 | 0.274 | 0.005 | 0.274 | 0.003 |
| VGG19 | 459.628 | 0.542 | 14.400 | 0.062 | 15 | 0.001 | 10 | 0.274 | 0.005 | 0.274 | 0.003 |
| VGG19 | 458.167 | 0.528 | 14.395 | 0.045 | 15 | 0.01 | 10 | 0.274 | 0.005 | 0.274 | 0.003 |
| VGG19 | 498.319 | 1.200 | 16.678 | 0.187 | 20 | 0.001 | 10 | 0.274 | 0.005 | 0.274 | 0.003 |
| VGG19 | 416.485 | 0.178 | 12.836 | 0.027 | 30 | 0.001 | 10 | 0.274 | 0.005 | 0.274 | 0.003 |
| VGG19 | 415.438 | 0.555 | 12.829 | 0.020 | 30 | 0.01 | 10 | 0.274 | 0.005 | 0.274 | 0.003 |
| VGG19 | 498.214 | 1.293 | 16.900 | 0.385 | 20 | 0.01 | 16 | 0.258 | 0.018 | 0.261 | 0.020 |
| ResNet152 V2 | 944.381 | 115.980 | 47.444 | 20.851 | 10 | 1.00E-05 | 1 | 0.885 | 0.004 | 0.997 | 0.001 |
| ResNet152 V2 | 920.769 | 246.532 | 27.395 | 0.259 | 15 | 1.00E-05 | 2 | 0.883 | 0.002 | 0.999 | 0.000 |
| ResNet152 V2 | 903.757 | 304.230 | 25.277 | 0.099 | 20 | 1.00E-05 | 3 | 0.878 | 0.007 | 0.999 | 0.000 |
| ResNet152 V2 | 688.470 | 0.368 | 25.204 | 0.117 | 20 | 0.0001 | 4 | 0.871 | 0.007 | 0.970 | 0.005 |
| ResNet152 V2 | 656.512 | 0.833 | 23.726 | 0.117 | 30 | 0.0001 | 5 | 0.866 | 0.012 | 0.968 | 0.012 |
| ResNet152 V2 | 761.664 | 146.595 | 23.697 | 0.156 | 30 | 1.00E-05 | 6 | 0.865 | 0.004 | 0.999 | 0.000 |
| ResNet152 V2 | 741.934 | 0.584 | 27.494 | 0.312 | 15 | 0.0001 | 7 | 0.862 | 0.009 | 0.965 | 0.007 |
| ResNet152 V2 | 857.165 | 1.070 | 32.996 | 0.237 | 10 | 0.0001 | 8 | 0.860 | 0.007 | 0.965 | 0.006 |
| ResNet152 V2 | 742.360 | 0.501 | 26.953 | 0.137 | 15 | 0.001 | 9 | 0.661 | 0.029 | 0.688 | 0.041 |
| ResNet152 V2 | 690.060 | 1.535 | 25.227 | 0.152 | 20 | 0.001 | 10 | 0.646 | 0.064 | 0.676 | 0.062 |
| ResNet152 V2 | 656.071 | 1.548 | 23.587 | 0.244 | 30 | 0.001 | 11 | 0.593 | 0.082 | 0.616 | 0.101 |
| ResNet152 V2 | 859.791 | 0.538 | 32.305 | 0.659 | 10 | 0.01 | 12 | 0.469 | 0.082 | 0.473 | 0.078 |
| ResNet152 V2 | 688.107 | 0.722 | 25.159 | 0.145 | 20 | 0.01 | 13 | 0.410 | 0.037 | 0.416 | 0.032 |
| ResNet152 V2 | 857.354 | 1.849 | 32.254 | 0.533 | 10 | 0.001 | 14 | 0.372 | 0.107 | 0.375 | 0.106 |
| ResNet152 V2 | 742.684 | 1.546 | 27.190 | 0.082 | 15 | 0.01 | 15 | 0.361 | 0.123 | 0.361 | 0.123 |
| ResNet152 V2 | 657.792 | 1.146 | 23.660 | 0.119 | 30 | 0.01 | 16 | 0.316 | 0.049 | 0.312 | 0.045 |
