# Supplementary material for: Deep learning algorithm for the automated detection and classification of nasal cavity mass in nasal endoscopic images
Source: PLoS One. 2024 Mar 13;19(3):e0297536. doi: 10.1371/journal.pone.0297536 (PMC10936791; doi:10.1371/journal.pone.0297536)
Supplement: S2 Table — (DOCX) [file pone.0297536.s004.docx]

**S2 Table. Grid of hyperparameters**

| **Batch size** | **Learning rate** |
| --- | --- |
| **10** | 0.01 |
| **15** | 0.001 |
| **20** | 0.0001 |
| **30** | 0.00001 |
