# Supplementary material for: Deep learning algorithm for the automated detection and classification of nasal cavity mass in nasal endoscopic images
Source: PLoS One. 2024 Mar 13;19(3):e0297536. doi: 10.1371/journal.pone.0297536 (PMC10936791; doi:10.1371/journal.pone.0297536)
Supplement: S3 Table — (DOCX) [file pone.0297536.s005.docx]

**S3 Table.** **Total number of trainable and non-trainable parameters of the CNN models**

| **CNN model** | **All Label Training** | |
| --- | --- | --- |
|  | **Trainable parameters** | **Non-trainable parameters** |
| **Xception** | 20,815,148 | 54,528 |
| **InceptionResNetV2** | 54,282,340 | 60,544 |
| **NasNetLarge** | 84,736,282 | 196,668 |
| **VGG19** | 246,541,380 | 0 |
| **ResNet152V2** | 58,196,100 | 143,744 |
